# Post-foraging in-colony behaviour of a central-place foraging seabird

**DOI:** 10.1038/s41598-022-17307-8

**Published:** 2022-07-28

**Authors:** Katarzyna Wojczulanis-Jakubas, Antoine Grissot, Marion Devogel, Lauraleen Altmeyer, Tessa Fujisaki, Dariusz Jakubas, Dorota Kidawa, Nina Karnovsky

**Affiliations:** 1 Department of Vertebrate Ecology and Zoology, Faculty of Biology, University of Gdańsk, Wita Stwosza 59, 80-308 Gdańsk, Poland; 2 French National Centre for Scientific Research-UMS 3343 OSUR, University of Rennes 1, Rue du Thabor, 35000 Rennes, France; 3 Department of Biology, Pomona College, 175 W. 6th, St. Claremont, CA 91711 USA

**Keywords:** Behavioural ecology, Animal behaviour

## Abstract

Studies on time allocation of various activities are crucial to understand which behavioural strategy is the most profitable in a given context, and so why animals behave in a particular way. Such investigations usually focus on a time window when the studied activity is performed, often neglecting how the time devoted to focal activity affects time allocation to following-up behaviours, while that may have its own fitness consequences. In this study, we examined time allocation into three post-foraging activities (entering the nest with food, nest attendance, and colony attendance) in a small seabird species, the little auk (*Alle alle*). Since little auks alternate foraging trips of different duration (short and long) and purpose (offspring feeding and primarily self-feeding, respectively) we expected that duration of the following up in-colony activities would also vary, being longer after a long absence in the colony (because of greater need of reassessment of the current predation pressure and social interactions in the colony, and re-establishing the bond with the offspring and/or partner and/or neighbours after longer absence). We found that it was not always the case, as time allocation of the post-foraging in-colony activities was primarily year- and sex-specific. It highlights the need to consider year and sex effects in studies of behavioural ecology, as not doing so may lead to spurious conclusions. Interestingly, and despite a great inter-individual variation in time allocation in the post-foraging in-colony activities, little auk individuals were quite repeatable in their behavioural performance, which suggests these activities may reflect birds behavioural profile. Overall, post-foraging in-colony activity of the little auk, although not much dependent on duration/type of the preceding foraging flights, varies with respect to year and sex, and as such may be a proxy of behavioural plasticity of the population.

## Introduction

Studies on time allocation in animals is of great importance to recognize behavioural mechanisms underlying basic life activities such as foraging or predator avoidance^1–4^. A thorough analysis of duration of particular activities in a given context is crucial to understand which behavioural strategy is the most profitable in terms of fitness, and the reasons why animals perform this particular way^5–7^. Most of the studies on time allocation only measure time windows of when a studied activity is performed. Considering foraging, for example, time intervals associated with particular foraging components (e.g. searching for food, latency to approach it, food handling and processing, etc.) are usually measured, and their relative durations are translated into foraging efficiency, e.g. Refs.^8–10^. What is often neglected, however, is how the time devoted to specific activities (e.g. foraging) affects time allocation to subsequent behaviours (e.g. resting). Time spent on such following-up behaviours, is not only important in the context of a total time budget but on its own may impact reproductive success and/or survival.

Numerous animals exhibit a central place foraging strategy during the breeding period, exploiting food resources away of a central location, i.e. nest or den, to which they are bound to return regularly^11,12^. When returning to its central location, a central place forager must reassess the situation there in terms of condition of the offspring and for many species also in terms of predation; it may also need to gather some information from conspecifics*.* Depending on the duration of the foraging trip, and what happened both in foraging areas and at the central location, the cost of such reassessment may vary, and so the time invested in various behaviours upon return may be allocated differently, which in turn, may have fitness consequences. For example, underestimating the predation risk upon return at the central place and entering the nest/den too early may cost the life of the adult or the brood.

Pelagic, colonial seabirds serve as good models to study behavioural consequences of post-foraging time allocation, as they perform regular foraging flights to distant foraging grounds carrying back food to their nestlings. Foraging at sea imposes potentially long absences from their nest, offspring and the breeding colony. To secure their own needs while caring for the offspring, some seabirds adopt a bimodal foraging strategy alternating foraging trips differing in duration: long and short^13,14^. During long trips breeding adults mainly rest and forage for self-maintenance, and only secondarily collect food for the offspring^13,14^. Short trips, on the other hand, serve primarily to collect food for the offspring, with no time spent on self-maintenance^15^. These two trip types thus impose not only different duration of the absence in the colony but also affect the nutritional state of individual^15^, which is obviously important when considering time allocation for post-foraging in-colony behaviour. While much attention has been given on understanding how these two types of trips differ in terms of duration, location, diving behaviour^16–20^ as well as the effect of environmental conditions on the bimodal foraging strategy (e.g. Refs.^21,22^), how allocation of time to foraging impacts the following up behaviour and time spent at the colony is poorly understood. Previous studies have shown that duration of colony attendance changes with respect to duration of foraging trips (e.g. Refs.^23–26^) but post foraging behaviour has not been examined explicitly.

Here we examined time allocation of post-foraging in-colony activities in the little auk (or dovekie) *Alle alle*, a small pelagic zooplanktivorous seabird, breeding colonially in the High Arctic^27^. Little auks breed in nest crevices in the scree of mountain slopes. Two parents care for a single egg/chick with equal contribution for most of the nesting period (only few days prior chick fledging the female stops feeding the chick and departs the colony, while the male continues to care for the chick and escorts the young in its first flight to sea^28–30^). Parents forage on Arctic zooplankton (mainly *Calanus* copepods^27,31^) and deliver it for the chick in a gular pouch, a special sack-like structure located under the bill. After returning from the foraging trip, little auks feed the chick (inside the nest chamber for most of the chick rearing and outside the nest at the end of the period)^27^. The little auk is a typical central place forager exhibiting a bimodal foraging strategy during the chick rearing period^16,32–34^. The short/long foraging trips are short or long both in terms of time and distance from colony; on average short trips last 1.3–3.9 h and are performed in a range up to 54 km from the colony, while long trips last 9.6–22.7 h, and span up to 150 km from the colony^16,17,20,35–37^. From both trip types, adults bring food for the offspring but feed themselves only during the long foraging trips^15^. Duration of short and long trips may vary across time and space^16,17,20,35–37^, sometimes with an increased duration of trips (especially long ones) in poorer environmental conditions (e.g. Ref.^16^), but the bimodal strategy during the chick rearing seems to be fixed^33,34^.

Little auks returning to the colony from a foraging trip face considerable risks of being predated, either by the glaucous gulls, *Larus hyperboreus*, or by the Arctic fox, *Vulpes lagopus*^27^*.* The former hunts primarily on adults present in or flying around the colony, while the latter hunts chicks and adults in the nest^38,39^. Both predators freely exploit the whole colony area and the location of nests with respect to the centre of the plot does not matter that much (*pers. obs*). Moreover, the returning adult often faces a complex social network of neighbours^27,40^. Handling this network may be even more important (e.g. for nest territory maintenance^40^) than interactions with the mate, as average nest density of 0.5–1.6 nests per m^2^ in the colony^41,42^ considerably increases probability of social interference while encounters of the breeding partners are not that frequent (*pers. obs.*). Finally, the returning adult is confronted with constant chick begging since the moment it enters the nest^43^. Thus, predation risk, social interactions and the needs of the chick could be factors to be considered by little auk breeders upon return from foraging. An under- or over-estimation of these factors may be costly, with a risk of death in the most extreme case. In this context, time allocation into post-foraging in-colony behaviours may be a good indicator of these challenges, and the way individuals cope with them.

The main aim of this study was to examine how little auks allocate their time in the colony after foraging with respect to type of the foraging trip (short vs long). We considered three main post-foraging in-colony behaviours: (1) latency to enter the nest, (2) nest attendance and (3) post-feeding colony attendance. We assumed these behaviours to be related to: (1) reassessment of predation pressure, (2) re-establishing the bond with the offspring and recognizing its current nutritional requirements^44^ and (3) re-establishing social status amongst closest neighbours^41^. Although assessment of the situation in the colony is likely to be performed after each episode of absence in a colony, we expected that long trips would lead to adults spending more time for each of the three behaviours compared to short trips. The reasoning behind this prediction was that reassessment of current level of predation risk may be more challenging after a long lasting absence. Similarly, re-establishing the social interactions may require more time after longer absences. Furthermore, after long foraging trips birds are also nutritionally satiated which is not the case after short trips^45^, thus they could devote more time for social interactions (with the offspring and neighbours).

## Materials and methods

### Field study

We carried out the study in the little auk breeding colony in Hornsund (SW Spitsbergen, 77° 00′ N, 15° 33′ E) in four consecutive breeding years (2017–2020). Each year, we captured both parents in focal nests (19 nests in 2017, 12 in 2018, 22 in 2019, and 18 in 2020) during the incubation period and marked them individually (alpha-numeric metal ring and colour combinations of plastic rings on both legs, and dyed breast feathers, made with a waterproof marker; Sharpie USA). We collected a feather sample for DNA-based sexing following a protocol described in Ref.^45^, when the first time a bird was captured. In total, we studied 91 different individuals, of which 42% were observed for more than one year (4 individuals followed for 4 years, 15 individuals for 3 years, 19 individuals for 2 years and 53 for a single year). Focal nests were distributed randomly across the colony plots; there were no apparent spatial outliers (i.e. all the nests were located in an area of similar nest density; see Supplementary Fig. S2). Given the way the main predators hunt for little auks (i.e. exploiting periphery and centre zones with similar rate, *pers. obs*.) we treated the nests as independent data points (i.e. not considering exact nest location with respect to the colony centre/periphery).

We recorded video of colony attendance and behaviour of marked individuals during the mid-chick rearing period (chicks age: 10–14 day of life). The mid-chick rearing was deliberately chosen because of the lowest probability of occurrence and/or frequency of potentially confounding behaviours (e.g. chick brooding, female brood desertion, pebble collecting, chick wandering out of the nest). The prevailing parental behaviour during this period is chick feeding performed inside the nest chamber, which greatly simplifies the analyses of activities selected for the present study. To establish hatching date (and consequently chick’s age), we checked the focal nests daily for the last week of incubation by a quick, visual inspection of the nest contents (egg/chick). Knowing the hatching date, we were able to plan the timing of video recording with respect to chicks age.

We recorded presence and behaviour of focal parents continuously in sessions for 72 h (2017 and 2020) or 48 h (2018 and 2019), with a single session performed for the nest in a given year. For recording we used commercial cameras (JVC R435REU, Japan) operating in time-laps mode (1 frame per sec). Each recording session allowed to capture on average 15.0 returns (range 2–62) from short trips that lasted on average 2.04 h (range 0.3–8.8 h, all years considered), and on average 2.5 returns (range 1–8) from long trips that lasted on average 15.6 h (range 6.7–29.4 h, all years considered) per individual per year, as well as the associated behaviours of interest performed after each trip. The time-laps resolution of 1 frame per sec, in combination with the duration of colony visit of parents (> 7 min; Grissot et al.^46^) was sufficient to detect the focal birds’ presence, recognize their identity, and characterize behaviours of interest.

Each nest was recorded with a dedicated camera, set up at ca 5–7 m of distance from the nest entrance/exit. We set the camera to cover a 6 m of diameter area, with the nest entrance/exit being in the centre of the frame. The majority of the focal nests had just a single entrance/exit, while only few of them had two, but in every cases the camera view was wide enough to capture all the nest openings. This configuration allowed us to spot focal birds when they were in the vicinity of the nest site within a 3 m radius around the nest as well as all entering in and exiting from the nest. Based on direct observations as well as data from GPS-tracked birds (*own unpublished data*), we assumed that this is where birds spent most, if not all, of their time while in the colony.

### Video processing

**Figure 1:**
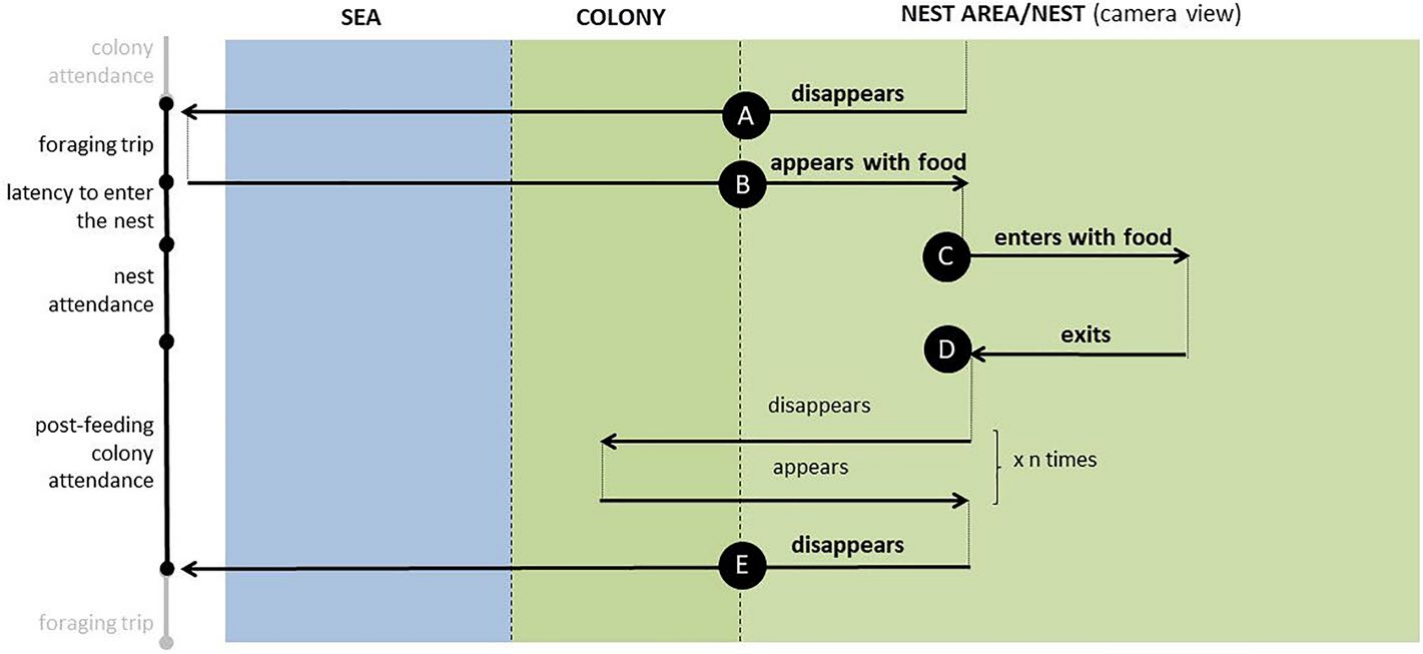
A scheme of video-analysis protocol. Letters in circles are key time-points for calculating the variables of interest (listed on the left side): the little auk parent disappears from the camera view for > 1 h (**A**,**E**) appears with food (**B**), enters the nest with food (**C**), and exits the nest after feeding (**D**).

We manually processed video material using VLC software (VideoLAN, France) or QuickTime player (Apple Inc. USA). We noted the time for each appearance and disappearance of a focal bird as well as the time it entered and exited its nest. We also noted information on whether the gular pouch was extended or not, indicating the presence or absence of food. Other events such as appearance of predators, direct interactions with neighbours and the presence of mate were also noted but being random and rare events were not considered in the present study. Based on these data, we calculated the duration of the foraging trips and post-foraging in-colony activities, termed here as latency to enter the nest, nest attendance (associated with feeding) and post-feeding colony attendance (hereafter colony attendance). We considered the duration of the foraging trip to be the time interval between a bird’s last observation in the nest area without food (“A” on Fig. 1), and its subsequent appearance with a food-full gular pouch (“B” on Fig. 1). Frequent disappearances and appearances without food, with time interval in between less than 30 min [often lasting up to 3 min], were not considered as foraging trips (but included into the time of the post-feeding colony attendance, see below). The absolute minimum for a foraging trip in the study area during the mid chick rearing period is 30 min after which, birds always return with food for the chick [established based on unpublished data from own observations and GPS tracks]. The short time when birds disappeared from the camera view were merely effect of either a predator appearance that scared the birds off from the colony plot, and/or the result of a too narrow camera view to capture rare events of an individual walking out the focal area. We considered latency to enter the nest to be the duration of a bird’s presence in the colony with food before entering the nest, following a given foraging trip, i.e. the time difference between the bird’s first appearance in the colony with food after completing the foraging trip (“B” on Fig. 1) and entering the nest (“C” on Fig. 1)]. For nest attendance (associated with feeding), we measured the time a bird spent in the nest chamber after arrival with food (for feeding the chick), i.e. the time difference between a bird’s entrance into the nest with food (“C” on Fig. 1) and exit without food (“D” on Fig. 1). We calculated post-feeding colony attendance after a given foraging trip based on the time difference between a bird’s exit from the nest after feeding (“D” on Fig. 1) and departure for the next foraging trip (“E” on Fig. 1). This colony attendance interval could include very short nest visits but since these were rare events and always short lasting (a typical pattern for mid chick rearing period), they were simply treated as a component of the colony attendance interval. All the short periods of birds absence due to predator or walking out of the camera view/frame (as described above) were also included in the post-feeding colony attendance.

### Data analysis

Given a clearly bimodal distribution of log-transformed duration of the foraging trips (regardless of year; Supplementary Fig. S4), we classified foraging trips as a short trip (ST) or a long trip (LT) following the method proposed by Welcker et al.^16^. With this method, the best cut-off value separates the trips in a way that minimizes the sum of variances of both trip types, given their log-normal distribution. We calculated the cut-off value separately for each year but the value was quite comparable for all studied years (2017: 7.0 h, 2018: 6.7 h, 2019: 8.8 h, 2020: 8.5 h, Supplementary Fig. S4). Even if the cut-off point was common for the 4 years (calculated on pooled data: 7.0 h), the results did not change qualitatively (see Supplementary Materials).

To examine how consistent are the birds exhibiting all the behaviours of interest (important for interpreting individuals effect and when averaging values for some further analyses), we estimated individuals’ repeatability of all in-colony activities (latency to enter the nest, nest attendance and post-feeding colony attendance) by applying linear mixed-effects models fitted by restricted maximum likelihood, implemented in *rpt* function in *rptR* package^47^ in R software^48^. Each post-foraging in-colony behaviour was considered in a separate model (response variable). Since we performed the analysis separately for data sets for ST and LT, we used the Gaussian error distribution. For both data sets, apart from bird identity being a random factor, we included year and sex as fixed variables in the model [thus looking at adjusted repeatability^47^]. We considered year effect, as each study year had distinct environmental characteristics (Supplementary Figs. S2 and S3) that affect little auks foraging performance (different durations of ST and LT, Supplementary Fig. S5) and so potentially also their post-foraging in-colony behaviours. In particular years, behaviours after LTs were less represented than those after STs, thus considering the year allowed also to control that bias (i.e. adding a year effect increased the range of confidence intervals for an estimate). We also included sex of the birds in the models due to possible sex-specific behavioural performance both in the colony [i.e., males spending more time in the colony^28,49^] and during foraging [i.e., females performing long trips of longer duration^16^].

To analyse the post-foraging in-colony activity and so to test the main hypothesis on the difference between birds behaviour after long and short trips, we first visually inspected the distribution of durations of all the three variables of interest: latency to enter the nest, nest attendance and post-feeding colony attendance. All the variables were strongly right skewed. Given that and the nature of the variable (the time elapsed since the onset of the behaviour) we analysed them with generalized linear models (*glm* function from basic R) using gamma error distribution. We analysed duration of each activity (response variable) in separate models, with type of foraging trip (ST/LT), year, sex and all their interactions as fixed factors [year and sex included in the model for the same reasons as described for repeatability analysis; the interactions were also included due to possible sex- and year- specific responses as observed in some other studies ^51^, Supplementary Fig. S5]. To account for pseudoreplication, we initially included bird identity as a random factor, however, when we did so, models did not converge. Therefore, to reduce the individual effect in such a data set, we averaged values for an individual for a given activity in a given year, and for each type of foraging trip separately. This approach resulted in single averaged values after LTs and STs in the given year and the given activity. Importantly, repeatability for most of the activities was different from zero (see “Results”), thus the averaged value used in the present analysis should properly characterize an individual. To report results of post-foraging in-colony activity models, we provided detailed model summaries and deviances in the tables, and plotted all the variables considered in the model (using ggplot2^52^). We did not test the inter-annual differences revealed in the results with post-hoc tests, as this was not relevant for the main topic of this study and would be analysed elsewhere.

We performed all the analyses in R 4.1.3 (R Core Team 2022). We considered results significant or marginally significant with alpha threshold of 0.05 and 0.1, respectively.

### Ethics approval and consent to participate

All applicable international rules for the use of animals, as specified in the guideline of the Association for the Study of Animal Behaviour, were followed. Besides birds were captured and marked under permission issued by the Norwegian Animal Research Authority (7/66141, 19/32026, 20/230619) and the Governor of Svalbard (17/00663-2, 17/00663-7, 20/00373-2).

## Results

**Figure 2:**
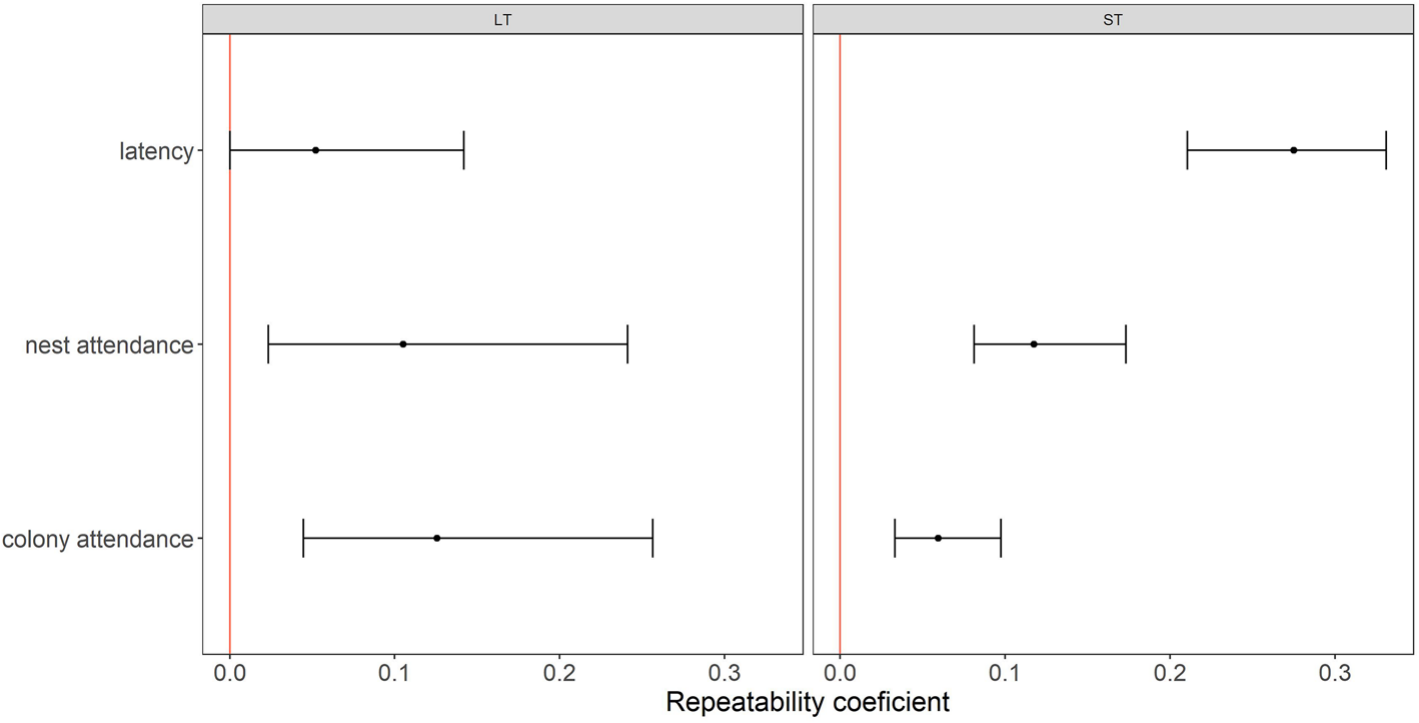
Size effect of repeatability adjusted for year and sex analysis for the three in-colony activities performed after long (LT) and short foraging trips (ST) by the little auk parents. Mean repeatability estimates (points) and its 95% confidence interval (CI, whiskers) are presented. CI range not overlapping with 0 (denoted with red vertical line) indicates significant effect at the alpha level of 0.05.

Duration of all in-colony behaviours after short trips (ST) were quite repeatable for individuals. The *R* values were not high (0.1–0.3) but in all cases their 95% confidence intervals were quite narrow and did not overlap with zero, meaning statistical significance (Fig. 2). After long trips (LT), both nest attendance and post-feeding colony attendance were also repeatable, though *R* values were quite low, with wide confidence intervals (Fig. 2). Only latency to enter the nest after LTs was not significant, with R < 0.1 and 95% confidence intervals overlapping with zero (Fig. 2).

**Figure 3:**
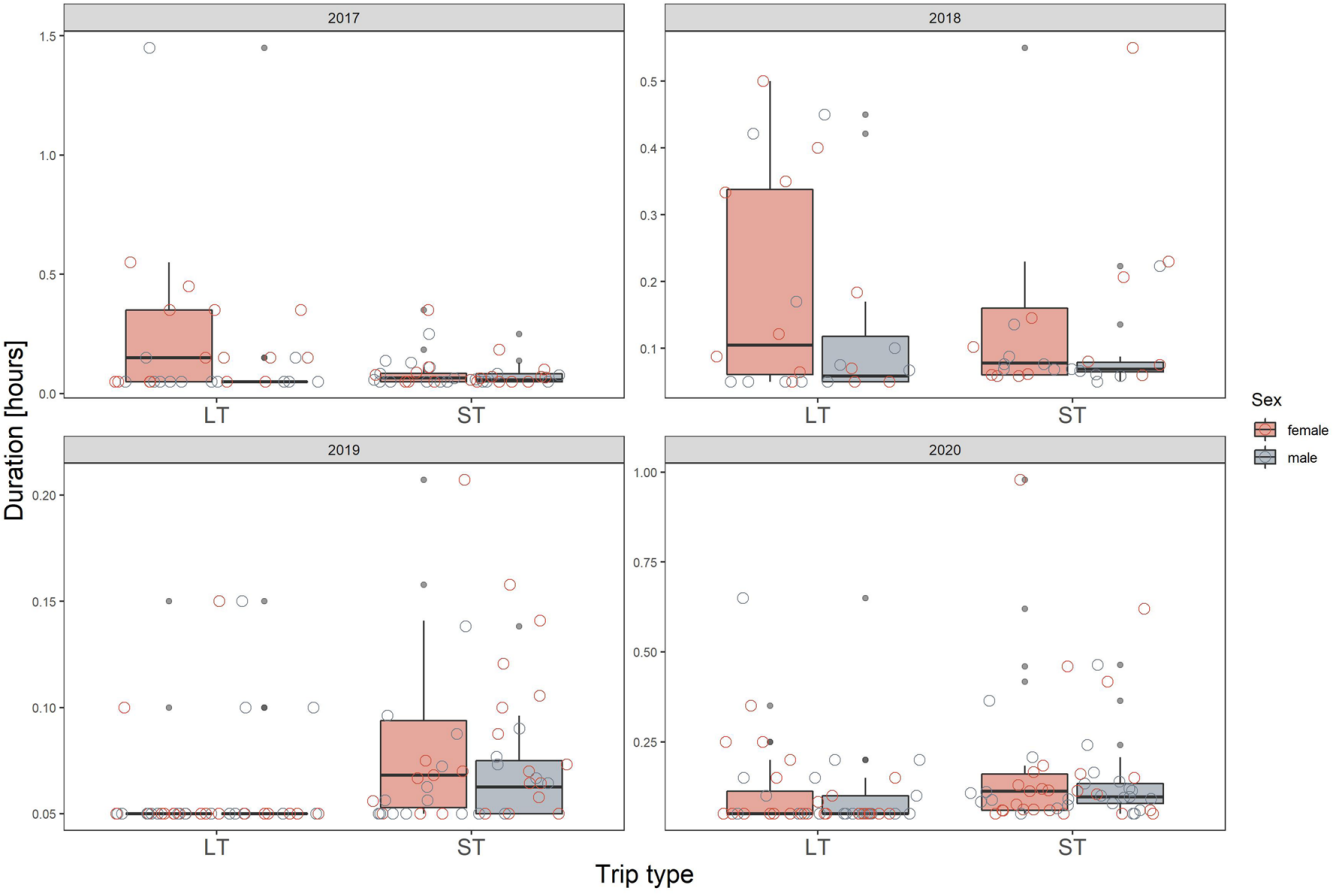
Latency to enter the nest with food after long (LT) and short foraging trip (ST) in respect to sex in 2017–2020. The scale on the y-axis is specific for the panel (year). Boxplots show the median (band inside the box), the first (25%) and third (75%) quartile (box), the lowest and the highest values within 1.5 interquartile range (whiskers) and outliers (dark grey dots). Empty circles present all the data points in each given group.

**Figure 4:**
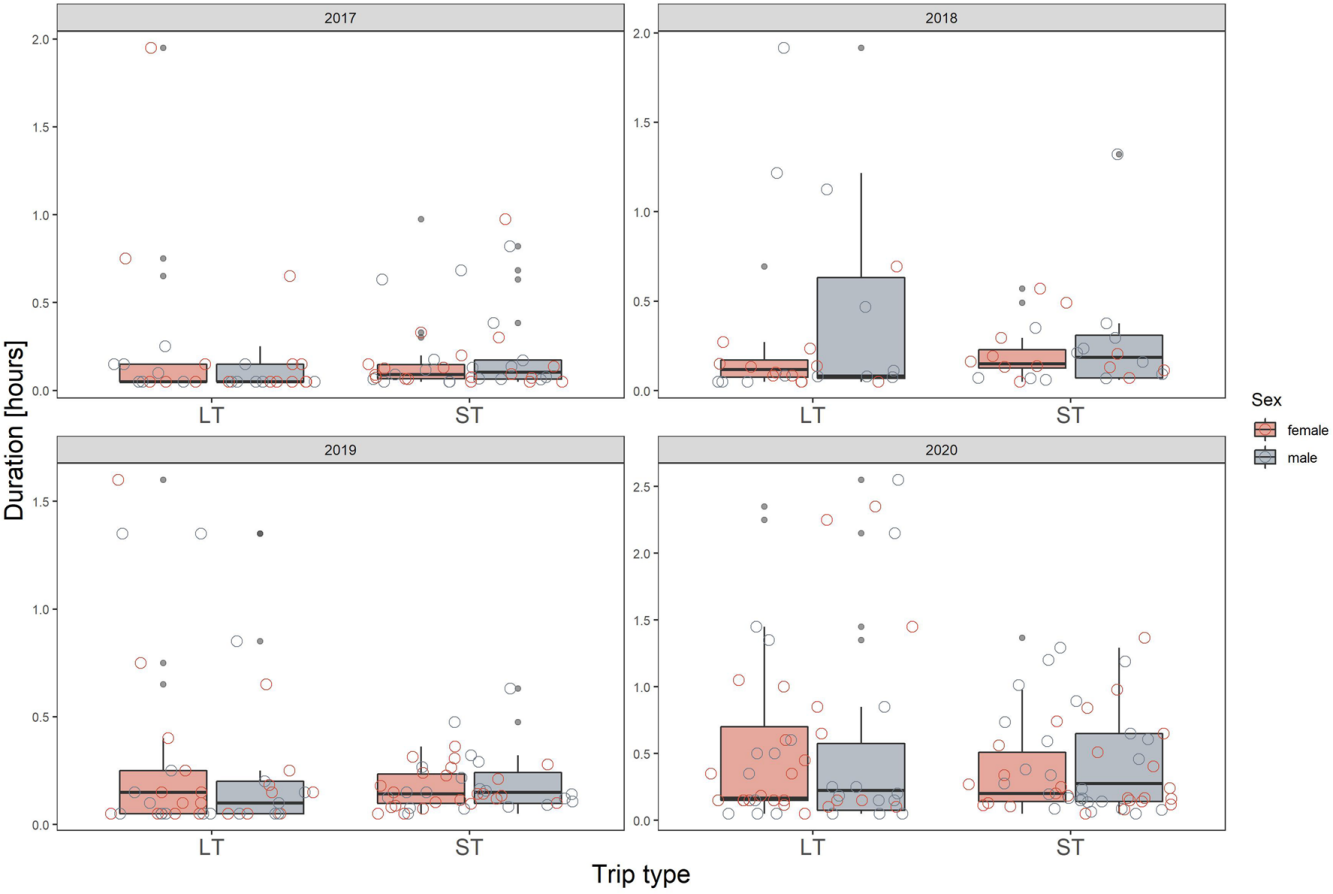
Nest attendance of little auks after completing long (LT) and short foraging trip (ST) in respect to sex in 2017–2020. The scale on the y-axis is specific for the panel (year). Boxplots show the median (band inside the box), the first (25%) and third (75%) quartile (box), the lowest and the highest values within 1.5 interquartile range (whiskers) and outliers (dots). Empty circles present all the data points in each given group.

**Figure 5:**
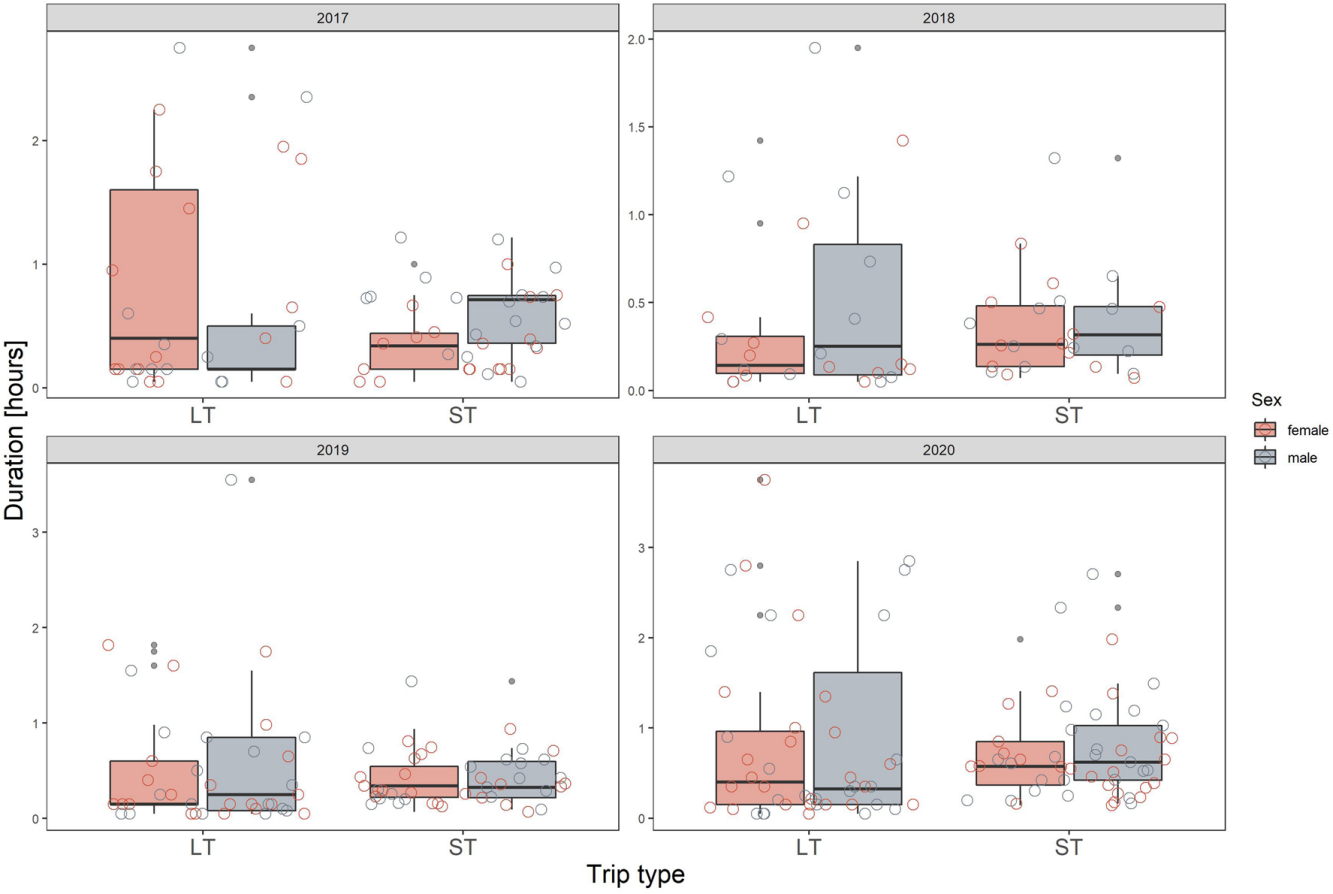
Colony attendance of little auks after completing long (LT) and short foraging trip (ST) in respect to sex 2017–2020. The scale on the y-axis is specific for the panel (year). Boxplots show the median (band inside the box), the first (25%) and third (75%) quartile (box), the lowest and the highest values within 1.5 interquartile range (whiskers) and outliers (dots). Empty circles present all the data points in given group.

We found that differences in latency to enter the nest after LTs and STs were primarily year-specific, both in magnitude and direction (Fig. 3, Table 1). In 2017 and 2018 the latency was longer after LTs than STs, as we originally expected, but observed the opposite in 2019 and 2020 (Fig. 3). The sex of the bird was not significant in this model, neither in its interaction with trip type nor year (Table 1, although overall, females tended to take more time to enter the nest than males (Fig. 3). Nest attendance was similar after completing STs and LTs, regardless of the year (Table 1), and although there were some sex- and year-specific responses, as indicated by significant interactions, they did not show any clear pattern (Table 1, Fig. 4). Colony attendance was greatly affected by the year and trip type but there was no consistent pattern (Fig. 5, Table 1). In all three behaviours considered, year was the most powerful factor explaining the observed variation in their duration (as indicated by the changes in deviance values, Table 2).

**Table 1:** Summary of generalized linear models (with gamma error distribution) describing post-foraging in-colony activities of little auks in regard to foraging trip type (ST/LT), sex (M/F) and year (2017–2020). Significant (p < 0.05), and marginally significant (p < 0.1) effects marked in bold and italics, respectively.

Term	Latency				Nest attendance				Colony attendance			
	Estimate	SE	Statistic	p value	Estimate	SE	Statistic	p value	Estimate	SE	Statistic	p value
Intercept	**5.08**	**1.23**	**4.13**	**< 0.001**	**3.53**	**1.06**	**3.33**	**0.001**	**1.83**	**0.53**	**3.46**	**0.001**
Trip type (ST)	**6.25**	**2.79**	**2.24**	**0.03**	2.42	1.94	1.25	0.21	**2.73**	**1.31**	**2.08**	**0.04**
Year 2018	0.23	1.89	0.12	0.91	2.36	2.24	1.05	0.29	**6.20**	**2.65**	**2.34**	**0.02**
Year 2019	**12.42**	**3.79**	**3.28**	**0.001**	0.45	1.46	0.30	0.76	**2.55**	**1.19**	**2.13**	**0.03**
Year 2020	**5.20**	**2.32**	**2.24**	**0.03**	− 1.70	1.14	− 1.48	0.14	**3.63**	**1.35**	**2.68**	**0.01**
Sex (m)	0.69	1.94	0.36	0.72	*7.30*	*3.65*	*2.00*	*0.05*	0.17	0.82	0.21	0.83
Trip type (ST) × year 2018	− 4.44	3.69	− 1.20	0.23	− 3.60	3.19	− 1.13	0.26	− 3.28	3.78	− 0.87	0.39
Trip type (ST) × year 2019	− **11.54**	**5.13**	− **2.25**	**0.03**	− 0.38	2.63	− 0.14	0.89	− 2.22	2.04	− 1.09	0.28
Trip type (ST) × year 2020	− **10.93**	**3.58**	− **3.06**	**0.002**	− 1.45	2.09	− 0.69	0.49	− *3.91*	*2.05*	− *1.91*	*0.06*
Trip type (ST) × sex (m)	0.39	4.20	0.09	0.93	− **8.54**	**4.20**	− **2.03**	**0.04**	− 1.85	1.64	− 1.13	0.26
Year 2018 × sex (m)	1.58	3.17	0.50	0.62	− **10.93**	**4.22**	− **2.59**	**0.01**	0.62	3.94	0.16	0.87
Year 2019 × sex (m)	− 2.00	5.50	− 0.36	0.72	− *7.81*	*3.91*	− *2.00*	*0.05*	− 0.95	1.66	− 0.57	0.57
Year 2020 × sex (m)	− 1.41	3.37	− 0.42	0.67	− *7.29*	*3.70*	− *1.97*	*0.05*	− 2.16	1.70	− 1.27	0.21
Trip type (ST) × year 2018 × sex (m)	1.78	6.12	0.29	0.77	**11.08**	**5.11**	**2.17**	**0.03**	0.84	5.37	0.16	0.88
Trip type (ST) × year 2019 × sex (m)	3.44	7.74	0.45	0.66	*8.18*	*4.86*	*1.68*	*0.09*	1.81	2.68	0.68	0.50
Trip type (ST) × year 2020 × sex (m)	2.46	5.33	0.46	0.64	7.96	4.33	1.84	0.07	3.29	2.56	1.29	0.20

**Table 2:** Deviances for all modelled post-foraging in-colony behaviours.

Parameters	Df	Df Resid	Latency		Nest attendance		Colony attendance	
			Deviance	Resid. Dev	Deviance	Resid. Dev	Deviance	Resid. Dev
Null		287		181.25		343.57		331.72
Trip type	1	286	0.33	180.92	5.87	337.70	2.25	329.47
Year	3	283	22.36	158.56	39.39	298.31	32.93	296.54
Sex	1	282	2.47	156.09	1.00	297.30	2.08	294.45
Trip type × year	3	279	16.21	139.89	1.41	295.89	3.92	290.54
Trip type × sex	1	278	0.71	139.18	0.69	295.20	0.41	290.13
Year × sex	3	275	0.89	138.29	6.04	289.16	0.66	289.47
Trip type × year × sex	3	272	0.25	138.04	7.67	281.50	2.20	287.27

## Discussion

Given the differences in duration of long (LT) and short foraging trips (ST), we expected that after LTs birds would spend more time in the colony before entering the nest with food compared to when they return from STs. We assumed that after a long time of being away from the colony they would need more time to recognize the current predation risk. Similarly, we expected birds to spend more time in the nest upon return from LTs, due to a need to re-establish the bond with the offspring and to reassess its needs. Since adults forage for their own needs only during LTs^44^, we assumed that after this type of trip, as they return satiated, they would stay longer in the colony. Our results show that this could be the case, however, apparently environmental conditions influence the post-foraging in-colony behaviours, and sex may further modify these behaviours. Our study highlights the fact that determinants of particular behaviours, when they are apparently conditions dependent, cannot be assessed based on a single sampling session. If we had carried out our study in a single year, we could have found either support for our hypotheses (e.g. longer latency to enter the nest after LTs compared to STS in 2018) or evidence for an entirely opposite pattern (e.g. shorter latency to enter the nest after LTs in 2019; Fig. 3, Supplementary Fig. S6).

Although the question of time-allocation after short and long trips has not been specifically examined before the present study, some seabirds species have been found to stay longer at the colony after long foraging trips, to coordinate nest attendance with their mates^24^ while other species do not^23^. Therefore it appears that factors which we have not considered in the present study may also play a role in shaping the overall time-budget of little auks. Since the aim of the present study was to compare little auk behaviour after long and short trips, we did not explicitly measure coordination with mates but this would be worth considering in future studies.

The inter-annual variability both in foraging as well as post-foraging in-colony behaviours suggests little auk behavioural plasticity. Adaptive changes in duration and frequency of foraging flights in respect to environmental conditions has been already demonstrated in the little auk, and all the results repetitively suggest that this species exhibits a high degree of flexibility in foraging strategies^17,35,53–58^. However, post-foraging colony behaviour has so far never been considered. Our results indicate that these behaviours also vary among years which probably reflects particular environmental conditions (to be examined in details in a separate study). These variable environmental conditions may affect predatory pressure, chick growth rate, begging behaviour intensity, and presence of conspecifics in the colony area, thus affecting duration of post-foraging behaviours, independently from foraging trip type. Given the flexibility of little auks in their foraging and post-foraging in-colony behaviours, future studies could seek to understand the particular environmental determinants of their allocation of time to the different behaviours, as well as the fitness consequences of a given time allocation.

Importantly, despite the great inter-individual variation (as expressed by inter-quartile ranges on all the boxplots, Figs. 3, 4 and 5) observed in the time allocation in post-foraging in-colony behaviours (which could be an effect of variables we could not control in the study, for example individuals age/experience) we found that duration of post-foraging in-colony behaviours was quite repeatable for individuals. This repeatability was lower for behaviours exhibited after LTs compared to STs which be due to smaller sample size and the year effect for the LTs data set (i.e. while there were multiple STs performed both within and among the years—on average 15 per individual per year, LTs were rarely represented more than twice per individual per year, and so the most of the repeatability in LTs originates from testing little auk behaviour across consecutive years; although the year was controlled in the analysis). Nevertheless, when considering repeatability of behaviours after STs only, it was in all cases significantly different from zero. One cause of repeatable behaviour could be the bird’s intrinsic characteristics, e.g. age, experience, etc. It is a possibility that latency to enter the nest, nest and colony attendance (when adjusted to sex and year) may be proxies of behavioural profile of the little auk, to be examined in future studies.

In conclusion, time allocation to post-foraging in-colony activity in the little auk does not change consistently in respect to duration of the foraging trip (short/long). It is primarily year-dependent, presumably linked to environmental conditions, and that in turn may be linked with differences in predatory pressure, chick growth or social conditions. Such results suggest behavioural plasticity of the species, exhibited not only in foraging strategies but also in post-foraging in-colony behaviours. Sex of the birds can further modify this time budget. Taken together this highlights the need to consider both year and sex when investigating the birds behaviour. Nevertheless, despite the great variation observed in the time allocation in post-foraging in-colony behaviours, little auks seem to be quite repeatable in duration of these behaviours, and that is a promising result for possible studies on, for instance, little auk personality and its effect on parental style reproductive success.

## Supplementary Information

Supplementary Information.

## Data Availability

Data and code for data analyses re accessible via the following Open Science Framework (OSF) repository: osf.io/vmxqk.
