# Author Correction: Post-foraging in-colony behaviour of a central-place foraging seabird

**DOI:** 10.1038/s41598-022-18895-1

**Published:** 2022-08-29

**Authors:** Katarzyna Wojczulanis-Jakubas, Antoine Grissot, Marion Devogel, Lauraleen Altmeyer, Tessa Fujisaki, Dariusz Jakubas, Dorota Kidawa, Nina Karnovsky

**Affiliations:** 1 Department of Vertebrate Ecology and Zoology, Faculty of Biology, University of Gdańsk, Wita Stwosza 59, 80-308 Gdańsk, Poland; 2 French National Centre for Scientific Research-UMS 3343 OSUR, University of Rennes 1, Rue du Thabor, 35000 Rennes, France; 3 Department of Biology, Pomona College, 175 W. 6th, St. Claremont, CA 91711 USA

Correction to: *Scientific Reports* 10.1038/s41598-022-17307-8, published online 28 July 2022

The original version of this Article contained an error in the spelling of the author Tessa Fujisaki which was incorrectly given as Tessa Fuijisaki.

The original Article and accompanying Supplementary Information file have been corrected.

