# Supplementary material for: Post-foraging in-colony behaviour of a central-place foraging seabird
Source: Sci Rep. 2022 Jul 28;12:12981. doi: 10.1038/s41598-022-17307-8 (PMC9334627; doi:10.1038/s41598-022-17307-8)
Supplement: Supplementary file 1 — Supplementary Information. [file 41598_2022_17307_MOESM1_ESM.docx]

**Supplementary materials**

**Post-foraging in-colony behaviour of a central-place foraging seabird**

Katarzyna Wojczulanis-Jakubas^1^*, Antoine Grissot^1^, Marion Devogel^1^, Lauraleen Altmeyer^1,2^, Tessa Fujisaki^3^, Dariusz Jakubas^1^, Dorota Kidawa^1^, Nina Karnovsky^3^

**Location of focal nests.**

All the nests considered in the study were randomly selected from all the nests that were available for researchers. Note that only part of the nests in a colony plot (unknown percentage) is available for the researchers due to nesting of the little auk in deep rock crevices. Also only part of the focal nest is denoted (due to missing coordinates for all the nests). The figure below illustrates spatial location of some of the focal (black crosses) and some of the other nests on the study plot. Importantly, the colony plot does not have any specific structure that would favour/ not favour some nests in respect to predation. Also the way the predators (the glaucous gull and Arctic fox) hunt on the little auk (exploiting freely the whole colony) does not favour/ not favour particular nests. All the focal nests were relatively close to each other (as denoted by the dashed lines of 49 m and 54 m long).


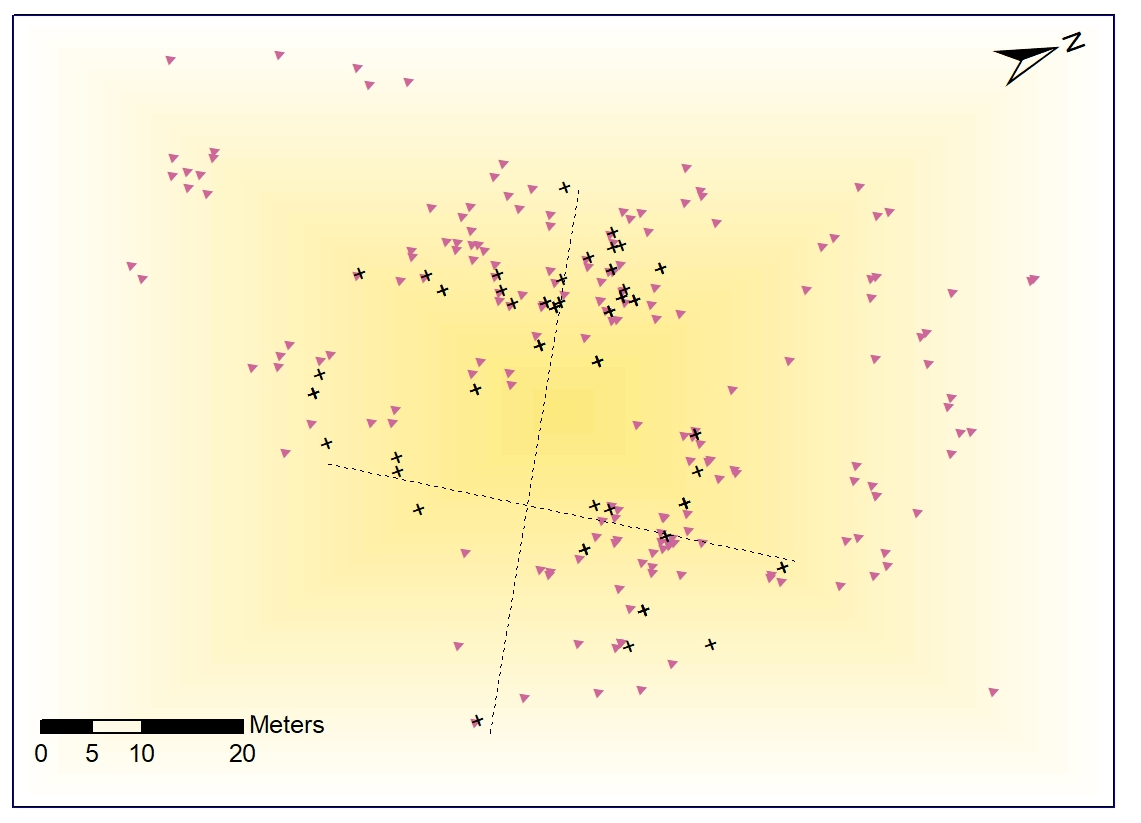


**Figure S1.** Spatial arrangement of focal (black crosses) and other nests (violet triangles) available to researchers in a colony plot. Dashed lines are to measure across-the plot distance (49 and 54 shorter and longer lines, respectively).

**Inter-annual variability of environmental conditions**

**Sea surface temperature** (SST) in July (the early and mid-chick-rearing period) was considered as a proxy of environmental conditions on the little auk foraging areas. SST has been recognized as important determinant of the occurrence of Arctic zooplankton, main prey items of the little auk on Svalbard (Kwasniewski et al. 2012; Carstensen et al. 2012).

To sample foraging areas of little auks in the Hornsund area we generated in ArcGIS software 10.3.1 (Redlands, CA, USA: Environmental Systems Research Institute) 500 random points within main feeding areas (95% kernel density) of chick-rearing individuals GPS-tracked in 2018 (Fig. S1A), see details in (Jakubas et al. 2020). We extracted SST for the random points in foraging locations of little auks from the Moderate-resolution Imaging Spectroradiometer (MODIS) Aqua satellite data. We used Level 3 daytime SST data derived from 11 μm thermal IR infrared (IR) bands with a 4 km spatial resolution from OceanColorData webpage (https://oceandata.sci.gsfc.nasa.gov/MODIS-Aqua). Then we compared SST values in random points between the studied years using Kruskal-Wallis and post-hoc Wilcoxon test for paired values using *ggpubr* package (Kassambara 2018) in R (R Core Team 2018).


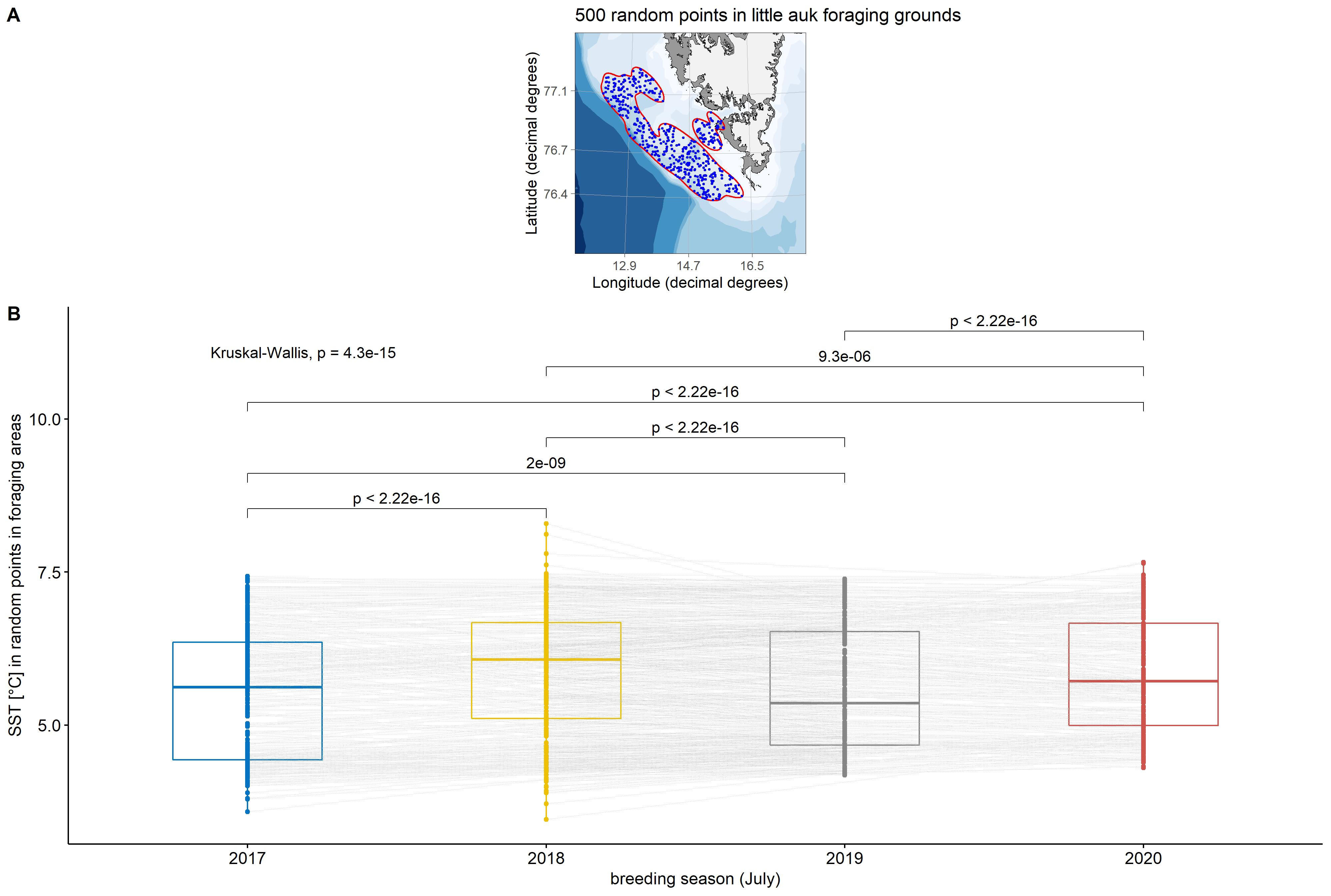


**Fig. S2.** **A.** Location of random points (blue points) generated within main foraging areas of little auks (expressed as 95% kernel density of foraging locations indicated with red polygon) breeding at Hornsund based on foraging position of chick-rearing individuals GPS-tracked in 2018. **B.** Sea surface temperature (MODIS-Aqua satellite data) in random points in July 2017, 2018, 2019 and 2020; lines with values represent p-values for Wilcoxon test for paired data. Boxplots show the median (band inside the box), the first (25%) and third (75%) quartile (box), the lowest and the highest values within 1.5 interquartile range (whiskers) and outliers (circles).

We found that all years differed significantly in SST values (Fig. S1B). The temperatures ranged from 3.46 to 8.29 ^o^C with the highest mean and median values recorded in 2018 (Fig. S1B).

**Meteorological parameters:** **air temperature, precipitation and wind speed** were considered as potentially influencing birds foraging and post-foraging in-colony behaviours. Air temperature and precipitation could impact brooding behaviour of little auk parents, with the behaviour being more frequently displayed, and lasting longer in colder and more humid conditions (due to chicks demands for brooding being higher in these conditions). The wind speed could affect birds foraging trips and/or colony attendance – in conditions of higher wind speed birds could spent more time to get to/from foraging areas/colony, and also could have spent less time in the colony (due to worse air-dragging during the little auk flight in the conditions of high wind speed).

All the three parameters were measured at Polish Polar Station that is located ca 1 km from the colony plot. The parameters were taken every three hours, and here we considered their daily averages, for the period from 20 to 31 July, which roughly covers the mid chick rearing phase in each year (i.e. when birds behaviour was investigated). Then, we analyzed the three parameters with Kruskal-Wallis and post-hoc Wilcoxon tests (separate for each parameter), using *ggpubr* package (Kassambara 2018) in R software (R Core Team 2018).

We found significant inter-annual differences in all the three parameters (Kruskal-Wallis tests, all p < 0.01, Fig. S3). Overall, of the four years, the warmest and the most windy was the year 2020, while the most humid was 2018 (Wilcox tests, p < 0.05, Fig. S3; see there also all the rest post-hoc comparisons).


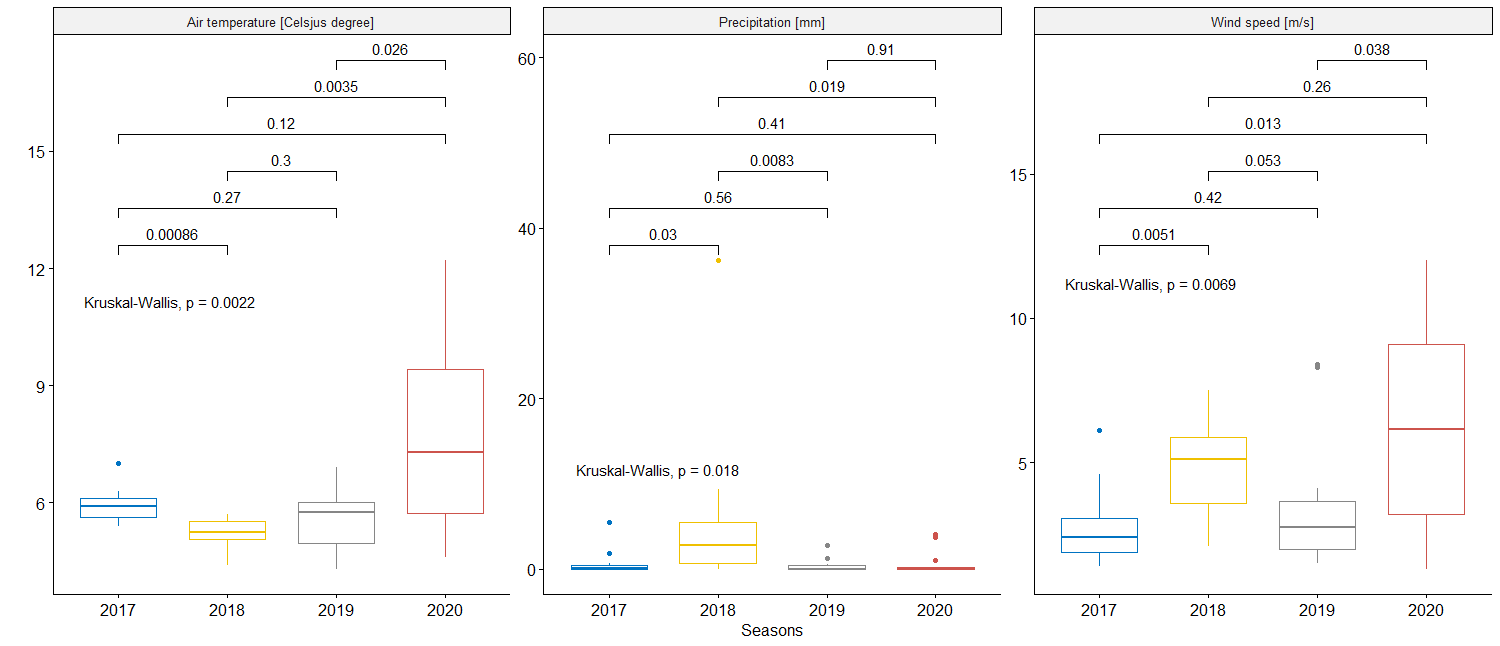


**Figure S3.** Inter-annual differences in meteorological parameters in Hornsund colony. Lines with values represent p-values for Wilcoxon test for paired data. Boxplots show the median (band inside the box), the first (25%) and third (75%) quartile (box), the lowest and the highest values within 1.5 interquartile range (whiskers) and outliers (circles).

**Inter-annual variability in the little auk foraging**

To divide foraging trips into ST and LT we applied method proposed by (Welcker et al. 2009). With this method, the best cut-off value separates the trips in a way that minimizes the sum of variances of both trip types, given their log-normal distribution. The cut-off values (either when calculated separately for each year or for all years being pooled) were quite similar: 2017: 7.0 h, 2018: 6.7 h, 2019: 8.8 h, 2020: 8.5 h and 7.0 h for pooled years. (Fig. S4).


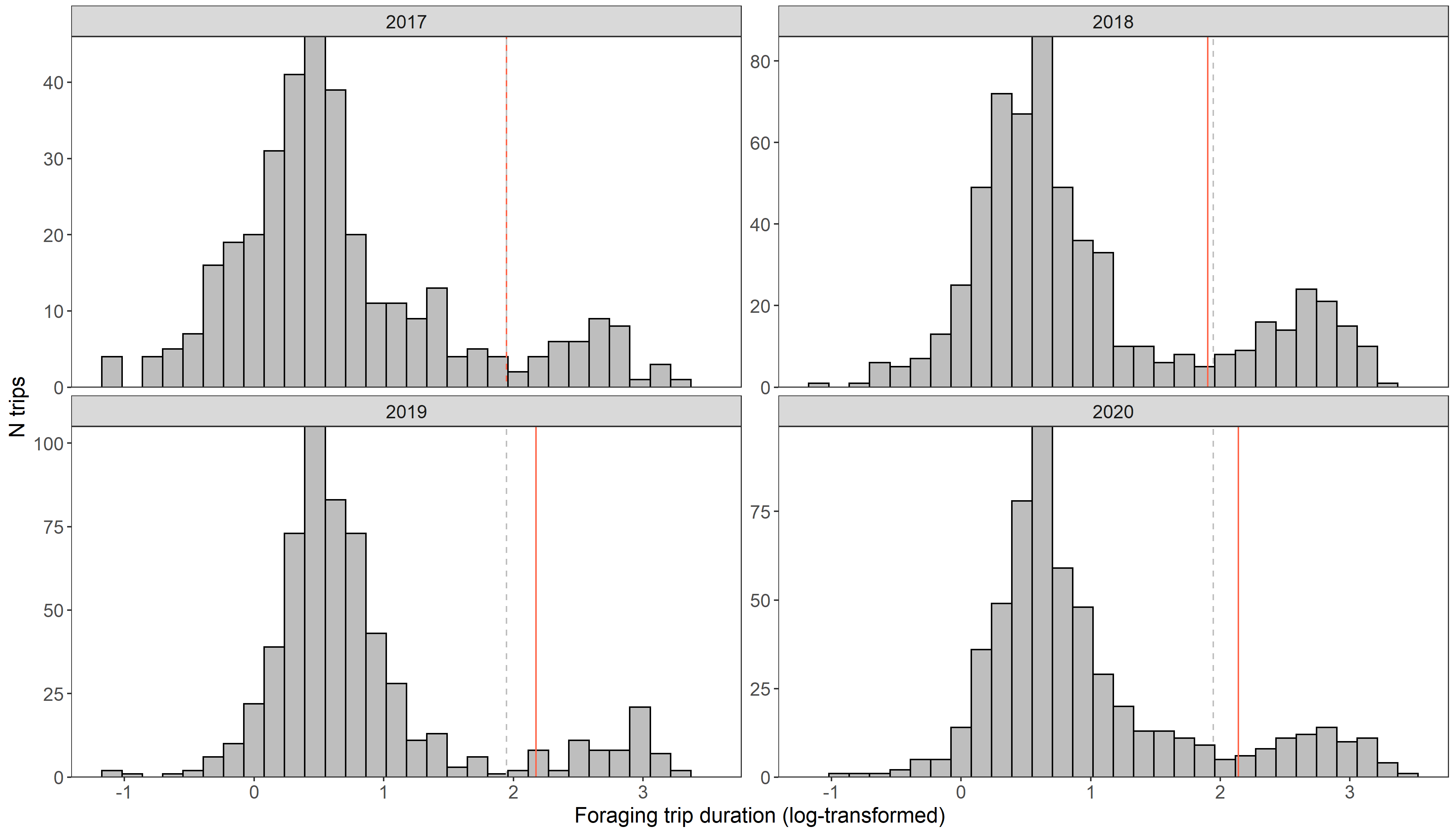


**Figure S4.** Log-normal distribution of little auk foraging trips (originally in lasting hours) in the four study years. Vertical lines denote cut-off values for the division for short (ST) and long trips; red-solid calculated separately for each year, grey-dashed calculated for all years being pooled.

To analyse inter-annual difference in duration foraging trips, for each trip type (ST/LT) separately, we applied linear mixed model fitted by restricted maximum likelihood (*lmer* function from *lmerTest* package, Kuznetsova et al., 2017), with log-transformed trip duration as a response variable and year and sex, as well as their interaction as fixed factors. We included in the model sex and its interaction with the year due to expected sex-specific foraging behaviour and possibly also sex-specific response to environmental factors (see the main text for justification). Since the same individuals were represented in the data set multiple times (the pseudoreplication especially relevant for ST data set but also LT when the same individual was followed for more than one year), we incorporated in the model birds’ identity as a random factor. The results were reported with analysis of variance table with Satterthwaite's method and basic characteristics (median, quantiles, range) were plotted in respect to significant variables considered in the model. Wilcox tests were performed as a post-hoc for inter-annual differences.

Despite similar ST/LT pattern over the study period (Fig. S4), we found inter-annual differences in duration of foraging trips both ST (LMM, F = 22.7, df = 3, P < 0.001), and LT data set (F = 5.19, df = 3, P = 0.001), although the differences were more apparent for the latter (Fig. S4). Although sex of birds was not significant for any data set (ST: F = 1.35, df = 1 P = 0.25; LT: F = 2.56, df = 1, P = 0.11), for ST sex interaction with year was significant (F = 6.13, df = 3, P< 0.001, but not for LT set: F = 0.76, df = 3, P = 0.52). That indicates on females performing slightly longer STs in some years than males (Fig. S5).


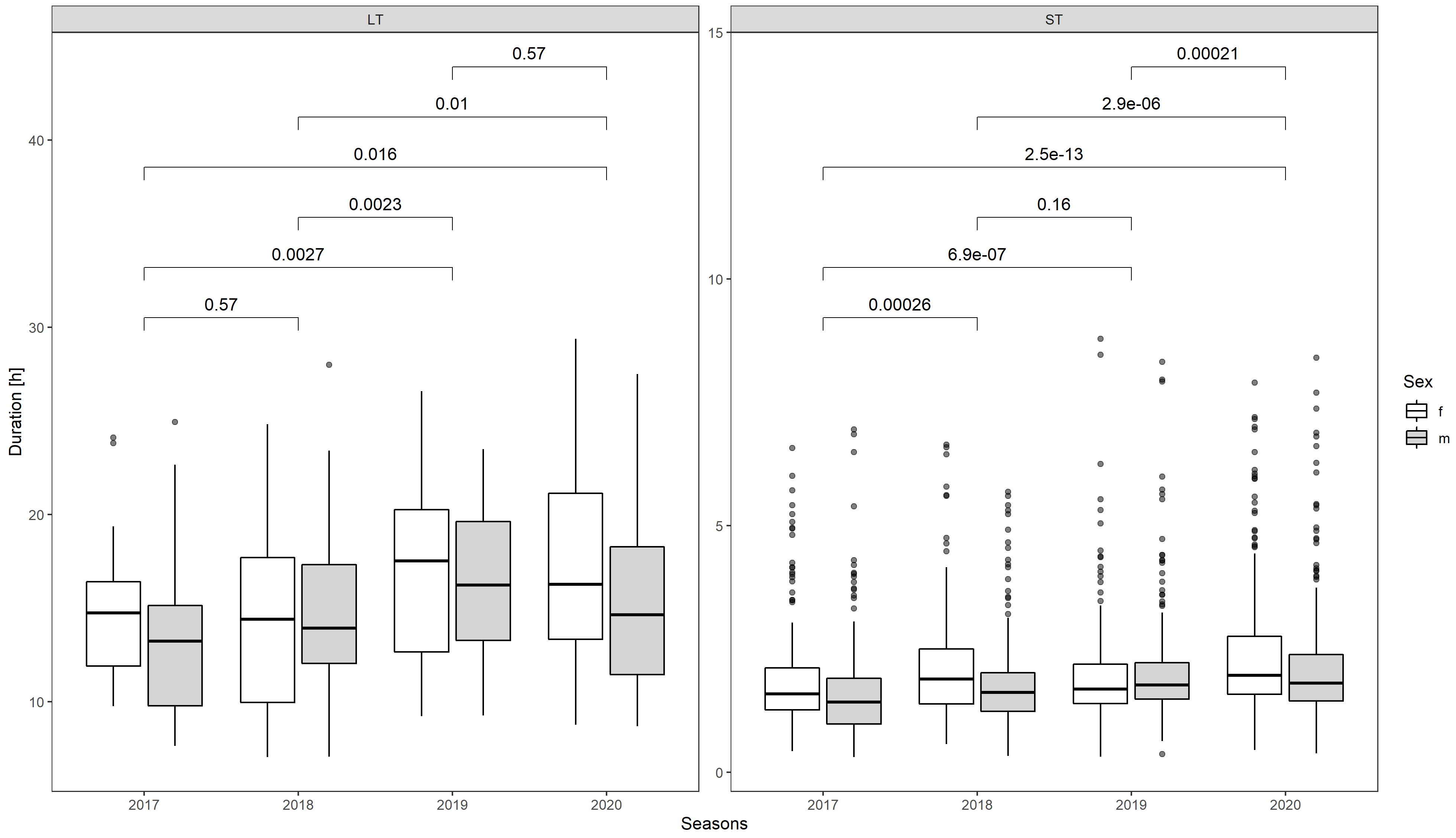


**Figure S5.** Duration (median – vertical line, 25-75% interquartile range – box, min-max vales excluding outliers – whiskers, and outliers – dots) of short and long foraging trips in respect to study year and sex.

**Results of the study (alternative approach to ST/LT division)**

Due to inter-annual variability in foraging conditions (Fig S2) and, as consequence, variation in little auk foraging behaviour (Fig. S4, and S5), we calculated the cut-off value separately for each year and presented in the main text. Nevertheless, the cut off values were quite similar for all the years (2017: 7.0 h, 2018: 6.7 h, 2019: 8.8 h, 2020: 8.5 h, Fig. S4) and its value calculated for pooled data was equal to 7.0 h. Since the way in which ST/LT are classified could potentially affect further results, we analysed all data using ST/LT division based on both year-specific and common cut-off values. The results did not differ qualitatively (difference in decimal points). We present the output based on ST/LT division with year-specific cut-offs in the main text, and here the alternative.

Duration of post-foraging in-colony behaviours were quite repeatable for individuals, except for latency after long trips, where 95% confidence intervals overlapped with zero (Fig. S6).


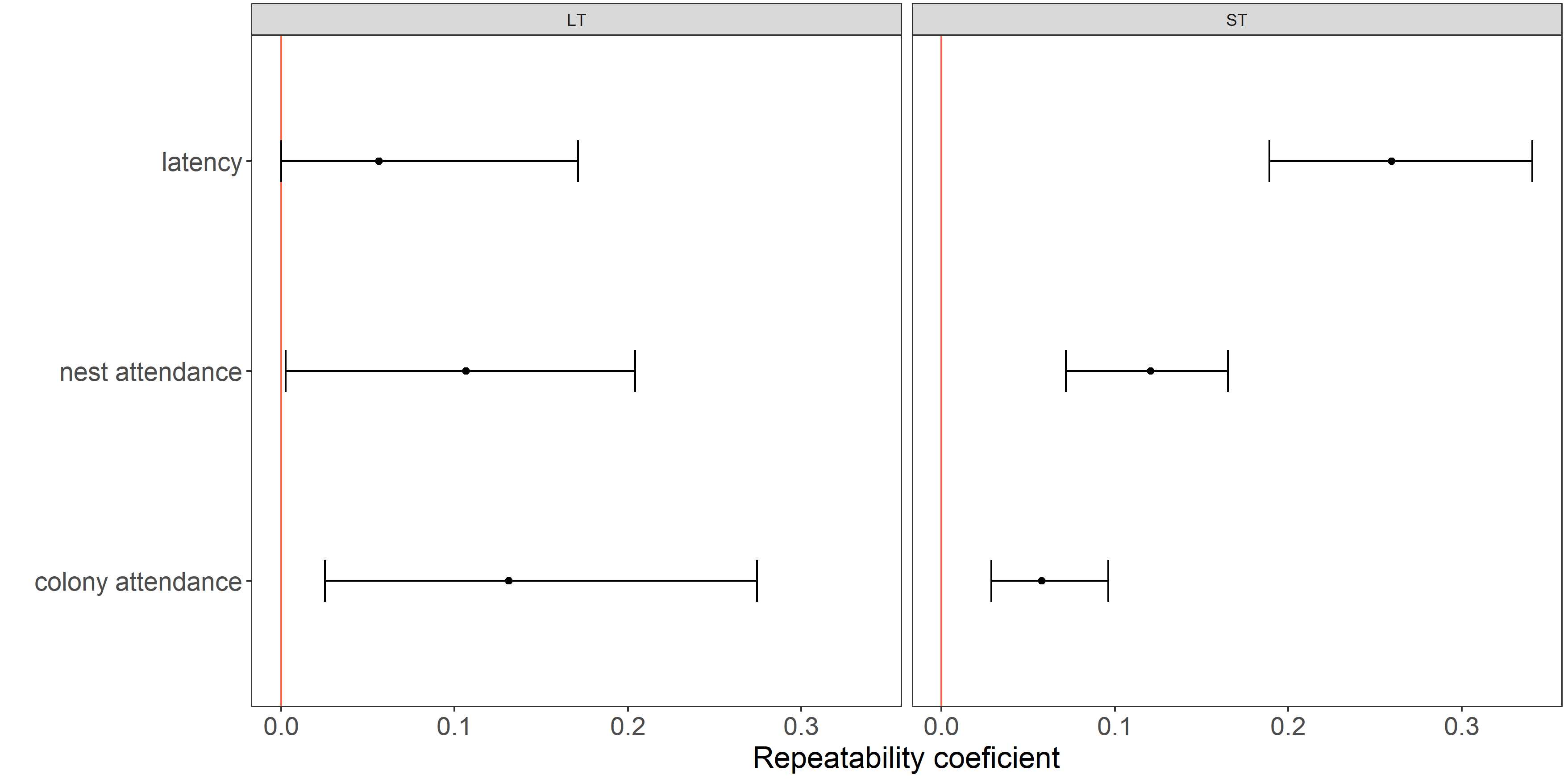


**Figure S6.** Size effect of repeatability analysis adjusted for year and sex for the three in-colony activities performed after long and short trips by the little auk parents. Mean repeatability estimates (points) and its 95% confidence interval (CI, whiskers) are presented. CI range not overlapping with 0 (denoted with red vertical line) indicates significant effect at the alpha level of 0.05.

Latency to enter the nest differed between LT and ST, and both magnitude and direction of the differences were year specific (Fig. S7, Table S1), in two years (2017 and 2018) latency was longer after LT than ST but in the other two years (2019 and 2020) the opposite was the case (Fig. S7). The sex, neither its interaction with trip type nor year were significant in the model (Table S1), although overall females tend to take more time to enter the nest than males (Fig. S7).


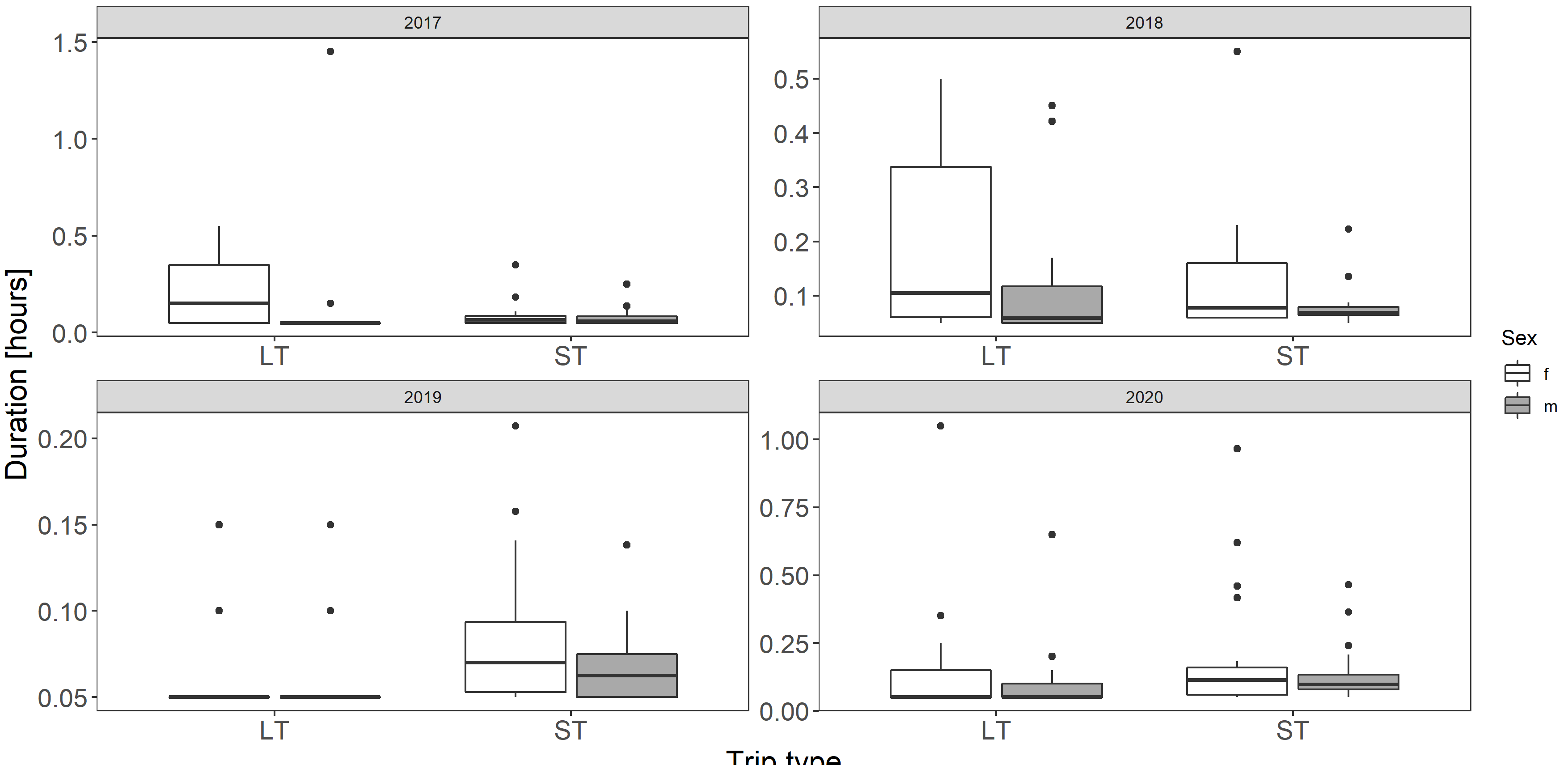


**Figure S7**. Latency to enter the nest with food after long (LT) and short trip (ST) in respect to parents sex in four study years. Note the scale on the y-axis is specific for the panel (year). Boxplots show the median (band inside the box), the first (25%) and third (75%) quartile (box), the lowest and the highest values within 1.5 interquartile range (whiskers) and outliers (circles).

Nest attendance was similar after completing ST and LT, regardless of year (Table S1). There were some sex- and year- specific responses, as indicated by sex, year and trip type interactions, however those do not make a clear pattern (Table S1, Fig. S8).


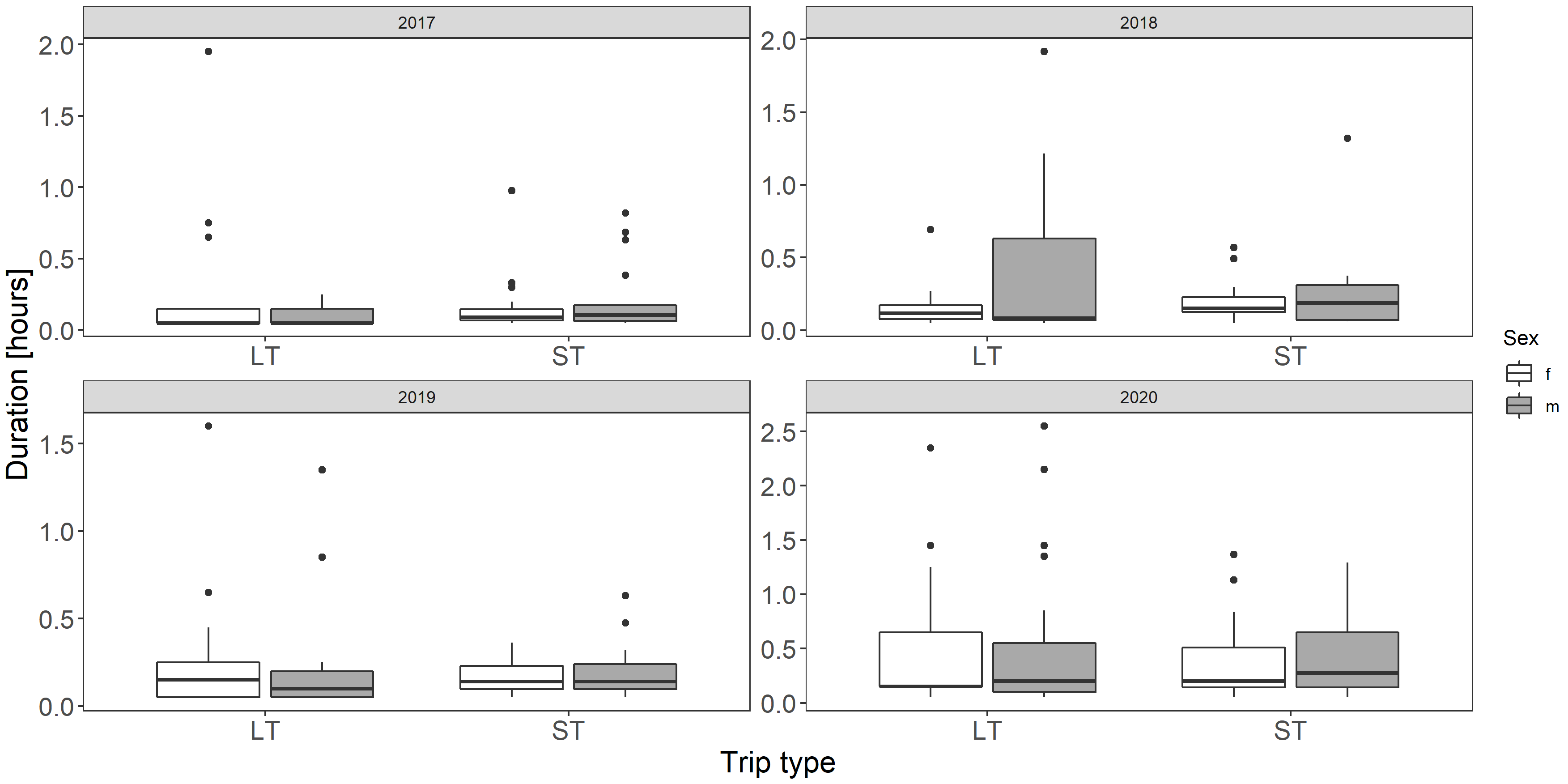


**Figure S8.** Nest attendance of little auks after completing STs and LTs in respect to parents’ sex in four study years. Note the scale on the y-axis is specific for the panel (year). Boxplots show the median (band inside the box), the first (25%) and third (75%) quartile (box), the lowest and the highest values within 1.5 interquartile range (whiskers) and outliers (circles).

Post-feeding colony attendance was similar regardless of the type of foraging trip performed, but apparently much affected by the year (Fig. S9, Table S1). Although sex was not significant in the model males tented to spend more time in the colony in some years (Fig. S9, Table S1).


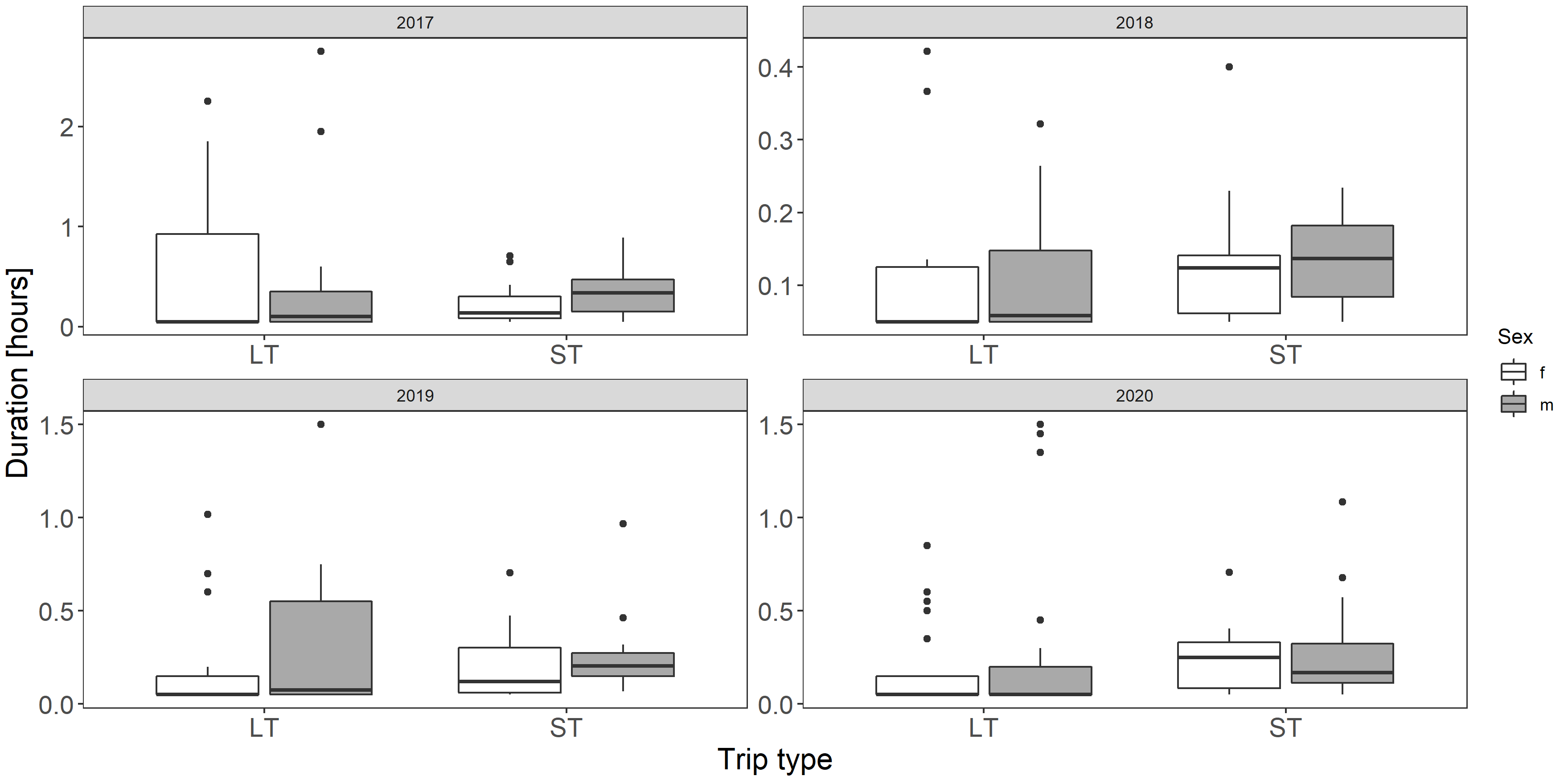


**Figure S9.** Post-feeding colony attendance of little auks after completing STs and LTs in respect to parents’ sex in four study years. Note the scale on the y-axis is specific for the panel (year). Boxplots show the median (band inside the box), the first (25%) and third (75%) quartile (box), the lowest and the highest values within 1.5 interquartile range (whiskers) and outliers (circles).

In all the three considered behaviours, year was the most significant factor explaining the observed variation, as indicated by the deviance (Table S2).

**Table S1.** Summary of LM models describing post-foraging in-colony activities of little auks in regard to foraging trip type (ST/LT), sex (M/F) and year (2017-2020). Significant (p < 0.05), and marginally significant (p < 0.1) effects marked in red and orange, respectively.

| **Term** | **Latency** | | | | **Nest attendance** | | | | **Colony attendance** | | | |
| --- | --- | --- | --- | --- | --- | --- | --- | --- | --- | --- | --- | --- |
|  | Estimate | SE | Statistic | P value | Estimate | SE | Statistic | P value | Estimate | SE | Statistic | P value |
| Intercept | **5.08** | **1.33** | **3.83** | **<0.001** | **3.53** | **1.06** | **3.33** | **0.001** | **1.83** | **0.53** | **3.47** | **0.001** |
| trip type (ST) | **6.25** | **3.01** | **2.08** | **0.04** | 2.42 | 1.94 | 1.25 | 0.21 | **2.73** | **1.31** | **2.08** | **0.04** |
| year2018 | 0.23 | 2.04 | 0.11 | 0.91 | 2.36 | 2.24 | 1.05 | 0.29 | **6.20** | **2.64** | **2.35** | **0.02** |
| year2019 | **12.42** | **4.08** | **3.04** | **0.003** | 0.61 | 1.49 | 0.41 | 0.68 | **3.09** | **1.31** | **2.36** | **0.02** |
| year2020 | 2.43 | 2.02 | 1.21 | 0.23 | -1.47 | 1.16 | -1.27 | 0.21 | **3.92** | **1.39** | **2.83** | **0.01** |
| sex (m) | 0.69 | 2.09 | 0.33 | 0.74 | **7.30** | **3.65** | **2.00** | **0.05** | 0.17 | 0.81 | 0.21 | 0.83 |
| trip type (ST) x year2018 | -4.44 | 3.97 | -1.12 | 0.26 | -3.60 | 3.19 | -1.13 | 0.26 | -3.28 | 3.77 | -0.87 | 0.38 |
| trip type (ST) x year2019 | **-11.57** | **5.53** | **-2.09** | **0.04** | -0.46 | 2.66 | -0.17 | 0.86 | -2.71 | 2.11 | -1.28 | 0.20 |
| trip type (ST) x year2020 | **-8.17** | **3.56** | **-2.30** | **0.02** | -1.73 | 2.10 | -0.83 | 0.41 | **-4.22** | **2.06** | **-2.04** | **0.04** |
| trip type (ST) x sex (m) | 0.39 | 4.52 | 0.09 | 0.93 | **-8.54** | **4.20** | **-2.03** | **0.04** | -1.85 | 1.63 | -1.14 | 0.26 |
| year2018 x sex (m) | 1.58 | 3.42 | 0.46 | 0.64 | **-10.93** | **4.22** | **-2.59** | **0.01** | 0.62 | 3.93 | 0.16 | 0.87 |
| year2019 x sex (m) | -2.00 | 5.92 | -0.34 | 0.74 | **-7.99** | **3.92** | **-2.04** | **0.04** | -1.74 | 1.71 | -1.02 | 0.31 |
| year2020 x sex (m) | 1.37 | 3.28 | 0.42 | 0.68 | **-7.45** | **3.71** | **-2.01** | **0.05** | -2.35 | 1.73 | -1.36 | 0.18 |
| trip type (ST) x year2018 x sex (m) | 1.78 | 6.59 | 0.27 | 0.79 | **11.08** | **5.11** | **2.17** | **0.03** | 0.84 | 5.35 | 0.16 | 0.87 |
| trip type (ST) x year2019 x sex (m) | 3.33 | 8.32 | 0.40 | 0.69 | **8.30** | **4.87** | **1.70** | **0.09** | 2.54 | 2.71 | 0.94 | 0.35 |
| trip type (ST) x year2020 x sex (m) | -0.36 | 5.53 | -0.07 | 0.95 | **8.17** | **4.33** | **1.89** | **0.06** | 3.50 | 2.57 | 1.36 | 0.17 |

**Table S2.** Deviance table for all modelling of post-foraging in-colony behaviours.

| Parameters | Df |  | **Latency** | | **Nest attendance** | | **Colony attendance** | |
| --- | --- | --- | --- | --- | --- | --- | --- | --- |
|  |  | Df Resid. | Deviance | Resid. Dev | Deviance | Resid. Dev | Deviance | Resid. Dev |
| NULL |  | 289 |  | 192.71 |  | 340.45 |  | 332.91 |
| trip type | 1 | 288 | 1.00 | 191.70 | 4.38 | 336.07 | 1.82 | 331.08 |
| year | 3 | 285 | 24.12 | 167.59 | 37.02 | 299.05 | 33.25 | 297.84 |
| sex | 1 | 284 | 3.73 | 163.85 | 1.47 | 297.57 | 2.81 | 295.02 |
| trip type x year | 3 | 281 | 12.99 | 150.86 | 1.61 | 295.96 | 4.45 | 290.58 |
| trip type x sex | 1 | 280 | 0.24 | 150.61 | 0.34 | 295.62 | 0.22 | 290.36 |
| year x sex | 3 | 277 | 0.62 | 149.99 | 5.94 | 289.69 | 1.10 | 289.26 |
| trip type x year x sex | 3 | 274 | 0.33 | 149.66 | 7.78 | 281.91 | 2.68 | 286.58 |

**References**

Carstensen J, Weydmann A, Olszewska A, Kwaśniewski S (2012) Effects of environmental conditions on the biomass of *Calanus* spp. in the Nordic Seas. J Plankton Res 34:951–966. doi: 10.1093/plankt/fbs059

Jakubas D, Wojczulanis-Jakubas K, Iliszko LM, et al (2020) Flexibility of little auks foraging in various oceanographic features in a changing Arctic. Sci Rep 10:8238. doi: 10.1038/s41598-020-65210-x

Kassambara A (2018) ggpubr: ‘ggplot2’ Based Publication Ready Plots. R package version 0.2.

Kuznetsova A, Brockhoff PB, Christensen RHB (2017) lmerTest Package: Tests in Linear Mixed Effects Models. J Stat Softw 82:1–26. doi: 10.18637/jss.v082.i13

Kwasniewski S, Gluchowska M, Walkusz W, et al (2012) Interannual changes in zooplankton on the West Spitsbergen Shelf in relation to hydrography and their consequences for the diet of planktivorous seabirds. ICES J Mar Sci 69:890–901. doi: 10.1093/icesjms/fss076

R Core Team (2018) R: A Language and Environment for Statistical Computing. R Foundation for Statistical Computing, Vienna, Austria.

Welcker J, Harding AMA, Karnovsky NJ, et al (2009) Flexibility in the bimodal foraging strategy of a high Arctic alcid, the little auk Alle alle. J Avian Biol 40:388–399. doi: 10.1111/j.1600-048X.2008.04620.x
